# Predictors of Gait Variability in Older Inpatients: An Exploratory Study Among Orthopedic Patients

**DOI:** 10.7759/cureus.71733

**Published:** 2024-10-17

**Authors:** Chen Xu, Yosuke Ishii, Masanosuke Mizutani, Takato Hashizume, Ryoichi Nakamura, Hiroshi Kurumadani, Makoto Takahashi

**Affiliations:** 1 Biomechanics, Graduate School of Biomedical and Health Sciences, Hiroshima University, Hiroshima, JPN; 2 Rehabilitation, Medical Technology, Shimura Hospital, Hiroshima, JPN; 3 Analysis and Control of Upper Extremity Function, Graduate School of Biomedical and Health Sciences, Hiroshima University, Hiroshima, JPN

**Keywords:** accelerometer, fall risk, gait variability, older inpatients, orthopedics

## Abstract

Aim

Older inpatients have reduced physical function and walking ability with a higher risk of falls after being discharged home. Gait variability can assess ambulation and is strongly related to the risk of falls. However, the clinical factors affecting gait variability in inpatients have not been identified. The purpose of this study was to investigate the predictive factors affecting gait variability in older inpatients.

Methods

A total of 42 older orthopedic inpatients with fractures of the hip, spine, and other segments and 18 healthy volunteers as the control group were enrolled in this study. Inpatients wore tri-axial accelerometers for a 10m walk before discharge. Gait variability was assessed by the coefficient of variation (CV) based on five consecutive stride times. Clinical assessment of muscle strength, joint mobility, balance, pain, and activities of daily living were also evaluated.

Results

The CV in inpatients was higher than that in healthy elderly. Quadriceps muscle strength, ankle dorsiflexion range of motion, and balance described the CV. When model 2 (adjusted R^2 ^= 0.473) was compared with model 1 (adjusted R^2 ^= 0.293), the quadriceps muscle strength and ankle dorsiflexion range of motion had a major effect on CV, while balance had not a greater influence than these two factors when compared with model 3 (adjusted R^2 ^= 0.537).

Conclusions

Poor knee extension strength, balance, and restriction of ankle dorsiflexion mobility have influenced gait variability in older inpatients.

## Introduction

Older patients who are hospitalized are commonly faced with functional decline [1]. During hospitalization, decreased movement performance, induced by a decline in physical function and walking ability, persists even after discharge [2,3]. Decreased movement performance can increase the risk of falls, leading to fractures and other injuries, resulting in readmission to the hospital [4]. Therefore, preventing falls due to decreased physical function and mobility is very important.

Gait variability is one of the risk factors for falls, which reflects fluctuations in strides as well as temporal variation. It provides vital information about the changes in gait rhythm during the gait cycle and is an effective indicator of gait ability [5]. By grasping some characteristics between strides, fall in the future can be effectively assessed and predicted [6]. Compared with spatiotemporal parameters such as average speed and average step length, gait variability is more sensitive to the identification of fall risk and more strongly related to falls, providing a basis for identifying fall populations [7-9]. A focus on gait variability may thus be valuable to support the quantification of fall risk.

Older inpatients have poorer physical function and mobility compared to healthy older adults. Disturbances from disease can lead to changes in gait patterns and variability, including increased fluctuations in each stride and irregular walking rhythms, which can also enhance the risk of falls [5,9]. Several studies have revealed that gait variability is influenced by various factors, such as lower extremity muscle strength, balance, etc. [6,10]. The risk of falls after discharge can be effectively reduced by targeting declining function with rehabilitation during hospitalization [11,12]. However, factors influencing gait variability on older inpatients have not been investigated.

We aimed to identify factors affecting gait variability in older inpatients and to explore the associated causes of falls. We hypothesized that factors related to motor function and easily trained for rehabilitation, such as lower extremity muscle strength and balance, would affect gait variability. This study is valuable for targeted training of inpatients to prevent falls after discharge.

## Materials and methods

Participants

A total of 42 older patients hospitalized for musculoskeletal disorders who could walk were selected for this study. The older inpatients included orthopedic patients with fractures of the spine, hip, and other segments around the hip joint due to falls in daily life. The general status of patients was divided from rank J (patients can go out independently) to rank A (patients can be independent indoors and go out with assistance) before hospitalization according to the Japanese version of the degree of independence in daily living for disabled older persons [13]. The control group consisted of 18 older healthy volunteers, each living independently in the community. Patients were excluded if they had neurological disorders, vestibular disease, cognitive impairment, or other conditions that can affect balance directly.

This study protocol was approved by the Institutional Review Board in Shimura Hospital (approval number: 19) and was conducted following the Declaration of Helsinki. All participants provided appropriate informed consent for participating in this study.

Clinical assessments

The clinical assessment includes function, ability, and factors that may impact gait variability. Inpatients are evaluated one week before discharge and the order of items was randomized. The assessments are shown below. Joint mobility of hip extension, knee extension, and ankle dorsiflexion was assessed using a universal goniometer to measure the maximum passive range of motion in a non-weight-bearing situation [14].

Quadriceps isometric muscle strength was measured by a hand-held dynamometer (HHD, μ Tas F-1, Anima Corporation, Tokyo, Japan) as a proxy for bilateral knee extension muscle strength [15]. The results of measurements were normalized according to body mass (N/kg). Besides, the 30-s chair stand test (CS-30) was also utilized to display the muscle strength of the lower extremities. Participants were seated in a 40 cm highchair with feet shoulder-width apart and arms crossed over the chest. They were then instructed to repeat stand and sit within 30 seconds. The score was the total number of stands a person can complete in 30 seconds, and incorrect movements such as not fully standing and sitting or using the upper limbs were not counted. The more times the participant completes it, the stronger the lower extremity muscles.

Weight-bearing ratio (WBR) was evaluated using two weight scales to record the pedaling force of each leg. The maximum value of each leg was divided by the weight as the lower limb load force [16]. Balance ability was determined using the Japanese version of the Mini-Balance Evaluation Systems Test (Mini-BEST). The Mini-BEST includes various static and dynamic balance items, providing a comprehensive assessment of the participant's balance ability. The score is rated on a 3-point scale from 0 to 2. The full score is 28 points. The higher the score, the better the patient's balance ability.

The degree of pain was assessed using the visual analog scale (VAS), which describes subjective perceptions of pain by participants. The functional independence measure (FIM) was used to reflect daily living activity levels and mobility limitations. High scores respond to more independent activities and daily performance.

Assessment of gait variability

Gait variability was calculated using accelerometers worn on the lower trunk during a 10-m walk [17]. The inertial sensors, including a tri-axial accelerometer and gyroscope sensor (WAA-010, ATR-Promotions, Japan), were attached over the lumbar spine at the level of L4 with a belt. During the walking process, the acceleration was collected at a sampling frequency of 100 Hz in three directions, anteroposterior, mediolateral, and vertical. Patients were asked to walk at a comfortable speed to reflect natural walking.

**Figure 1:**
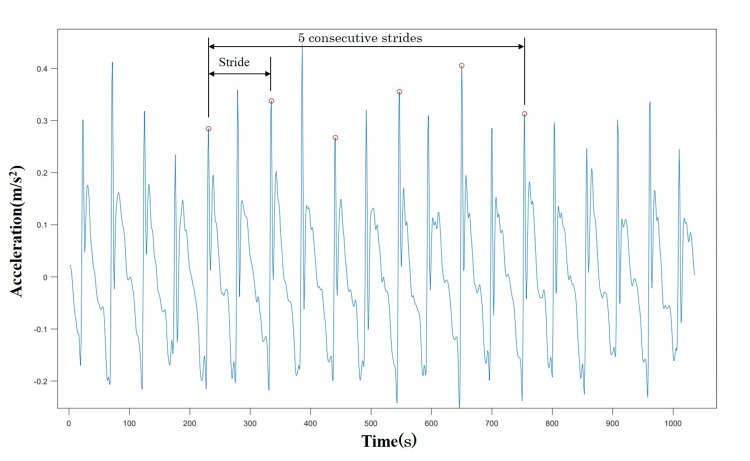
An example of inpatient acceleration in the anteroposterior direction The red circle indicates the peak of acceleration, defined as the moment of initial contact with five consecutive strides.

All data were analyzed using MATLAB software (MATLAB 2022a, MathWorks, Japan). First, all the raw acceleration data are smoothed with high- and low-pass filters at cutoff frequencies of 0.2 and 20 Hz, respectively. The stride duration was determined based on the moment at the heel contact during steady walking [18]. The moment coincides with the peak in the anterior-posterior direction of the accelerometer signals. A stride is defined as the time interval between one peak and the next (Figure 1). Gait variability was defined as the coefficient of variation (CV) of five consecutive strides durations and was calculated as the formula (standard deviation of step duration (SD)/mean step duration) × 100.

Statistical analyses

The demographic data and gait parameters of patients and control groups using the independent t-test, and stride time using the Mann-Whitney U test for systematic differences. Regarding the clinical assessment of different fracture sites, the Shapiro-Wilk test was used to test that FIM, VAS, knee extension range of motion (ROM), ankle dorsiflexion ROM, and stride time did not conform to the normal distribution, and these were analyzed using the Kruskal-Wallis test. Gait variability and other clinical assessments of inpatients using one-way analysis of variance. To determine the factors associated with gait variability, stepwise multiple regression analysis was performed with the stride time CV as the dependent variable and clinical assessment, such as age, BMI, hip extension ROM, knee extension ROM, ankle dorsiflexion ROM, QS, CS-30, WBR, Mini-BEST, VAS, and FIM as independent variables. SPSS (v24, IBM Corp., Armonk, NY, USA) was used for statistical analysis, with the significance level set at P < 0.05.

## Results

Participants’ demographic data

A total of 60 participants were enrolled in our study, and their demographic data are shown (Table 1). There were significant differences in age between inpatients and healthy older adults. Inpatients contained multiple fracture sites, including 24 (57%) of hip fractures, 13 (31%) of spine fractures, and five (12%) of other fractures around the hip joint. The history of falls was present in 14 (33%) of hip fracture inpatients, six (14%) of spine fracture patients, and two (4%) of other fracture inpatients. Besides, there were 25 (60%) of inpatients with rank J1, eight (19%) with rank J2, eight (19%) with rank A1 and one (2%) with A2.

**Table 1:** Demographic data of participants BMI, Body mass index. The values of Age and BMI represent the means ± standard deviation. * denotes a significant difference between inpatients and healthy individuals (p<0.05).

	Inpatients	Healthy	t-value	P-value
Participates, N	42	18	-	-
Gender, Male/Female	12/30	10/8	-	-
Age (years)	77.98±10.22	66.33±4.34	4.63	<0.01*
BMI (kg/m^2^)	21.74±2.64	22.86±1.61	-1.66	0.10
Fracture site, N (%)				
Hip	24(57)	-	-	-
Spine	13(31)	-	-	-
Other	5(12)	-	-	-
Fall history, N (%)				
Hip	14(33)	-	-	-
Spine	6(14)	-	-	-
Other	2(4)	-	-	-
Degree of independence in daily living for disabled elderly persons, N (%)				
J1	25(60)	-	-	-
J2	8(19)	-	-	-
A1	8(19)	-	-	-
A2	1(2)	-	-	-

Clinical assessments

The comparison of clinical assessments in patients with hip, spine, and other fracture sites is reflected (Table 2), and there were no significant differences among various fracture sites.

**Table 2:** Clinical assessments at different fracture sites ROM, range of motion. QS, isometric quadriceps muscle strength. CS-30, the 30-s chair stand test. WBR, Weight Bearing Ratio. Mini-BEST, Mini-Balance Evaluation Systems Test. VAS, visual analog scale. FIM, functional independence measure. The knee extension ROM, ankle dorsiflexion ROM, FIM and VAS were analyzed using the Kruskal-Wallis H-test, while other parameters were analyzed using the F-test of one-way ANOVA. The values represent the means ± standard deviation. The p-value relates to comparisons of hip, spine, and other fracture sites (p<0.05).

	All inpatients (N=42)	Hip (N=24)	Spine (N=13)	Other (N=5)	F/H-value	P-value
ROM (°)						
Hip extension	13.88±6.45	14.57±6.89	12.50±5.84	14.00±6.52	1.18	0.32
Knee extension	3.87±5.83	5.22±5.93	1.25±5.28	4.00±5.48	1.77	0.41
Ankle dorsiflexion	14.13±5.18	15.43±4.75	12.92±5.42	11.00±5.48	2.61	0.27
QS(N/kg)	2.51±0.95	2.34±0.76	2.69±1.15	2.88±1.25	0.86	0.43
CS-30(number of times)	10.63±4.40	10.83±4.73	9.92±4.14	11.40±3.98	0.20	0.82
WBR (%)	81.90±6.91	82.00±6.02	82.00±9.07	81.20±6.30	0.03	0.97
Mini-BEST (score)	19.35±3.79	19.48±3.90	18.75±3.98	20.20±3.27	0.32	0.73
VAS (0-10)	1.15±1.17	1.33±1.05	0.92±1.45	0.88±1.03	1.49	0.47
FIM (score)	120.20±8.47	120.57±5.86	117.83±13.00	124.20±2.68	3.05	0.22

Gait parameters

**Figure 2:**
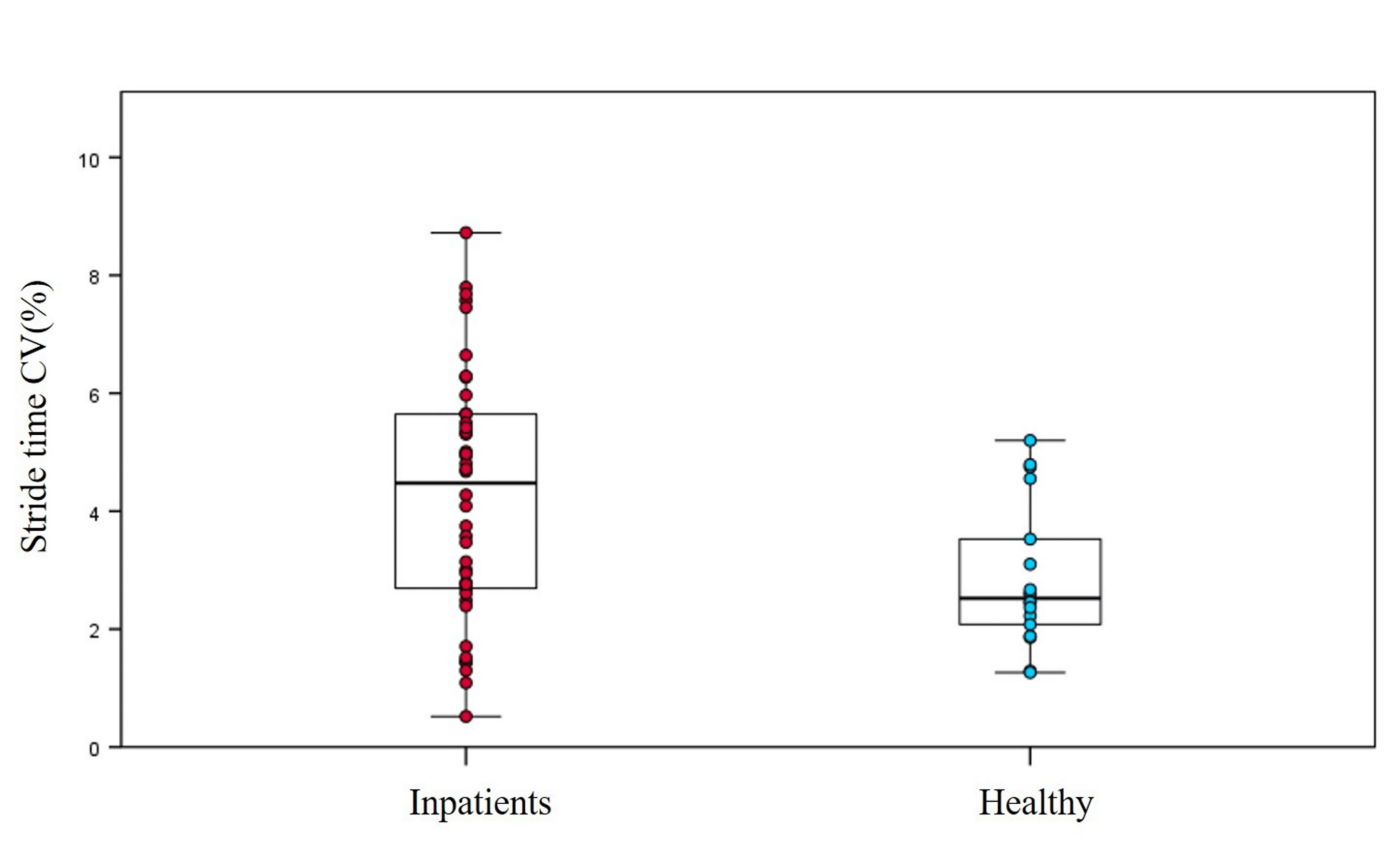
Characteristics of stride time CV between inpatients and healthy older adults CV, coefficient of variation. The scatter represents the dispersion of stride time CV in participants. For each box, the interior line in bold shows the median, and the edges of the box are estimates of the first and third quartiles. p<0.05

Comparisons of gait parameters between inpatients and healthy individuals and subgroups are shown (Table 3). Inpatients have longer stride time, slower gait velocity, and lower cadence than healthy older adults. The stride time CV between inpatients and healthy older adults showed significant differences (Figure 2). The stride time CV of inpatients was higher than healthy older adults (inpatients: 4.3 ± 2.1%, healthy older adults: 2.9 ± 1.2%, p<0.01).

**Table 3:** Comparisons of gait parameters between inpatients and healthy individuals and subgroups of patients with fractures at each site Stride time CV, Stride time Coefficient of Variation. Comparisons between inpatients and healthy individuals for stride time utilized the Z-value of the Mann-Whitney U test, and other parameters utilized the t-value of the t-test. Comparisons among inpatients with injuries at each fracture site for stride time were analysed using the Kruskal-Wallis H-test, while other parameters were analysed using the F-test of one-way ANOVA. The values represent the means ± standard deviation. * denotes a significant difference between inpatients and healthy individuals (p<0.05).

	All inpatients (N=42)	Healthy (N=18)	Z/t-value	P-value	Hip (N=24)	Spine (N=13)	Other (N=5)	H/F-value	P-value
Stride time (s)	1.15±0.21	0.99±0.08	-3.11	<0.01*	1.17±0.21	1.13±0.22	1.12±0.23	0.80	0.67
Gait velocity (m/s)	0.83±0.26	1.29±0.26	-6.21	<0.01*	0.78±0.24	0.89±0.24	0.93±0.39	1.23	0.30
Cadence (step/min)	106.97±17.05	122.02±10.60	-4.15	<0.01*	105.04±16.98	109.21±17.08	110.45±19.80	0.35	0.70
Stride time CV (%)	4.27±2.08	2.87±1.21	3.26	<0.01*	4.16±2.06	4.55±2.19	4.06±2.33	0.16	0.85

Factors associated with gait variability using multiple regression analysis

The results of the multiple regression analysis are shown in Table 4. The independent variables identified as Stride time CV were QS, ankle dorsiflexion ROM and Mini-BEST. The effect of QS and ankle dorsiflexion ROM on stride time CV was stronger than the effect of QS as a single factor when comparing model 2 (adjusted R^2^ =0.47) with model 1 (adjusted R^2^ =0.29), while the effect of balance did not play a major role when comparing model 2 with model 3 (adjusted R^2^ =0.54).

**Table 4:** Stepwise multiple regression analysis for Stride time CV as the dependent variable in all patients QS, isometric quadriceps muscle strength. ROM, range of motion. Mini-BEST, Mini-Balance Evaluation Systems Test. CI, confidence interval. CV - coefficient of variation. Model 2 was adjusted for ankle dorsiflexion ROM using a general linear regression model. Model 3 was adjusted for ankle dorsiflexion ROM and Mini-BEST using a general linear regression model. * denotes a significant difference (p<0.05).

Model	Variables	Standard β	P-value	95% CI	R	R^2^	Adjusted R^2^
1	QS (N/kg)	-0.56	<0.01*	-1.91 to -0.62	0.56	0.31	0.29
2	QS (N/kg)	-0.48	<0.01*	-1.65 to -0.51	0.71	0.50	0.47
	Ankle dorsiflexion ROM (°)	-0.44	<0.01*	-0.28 to -0.08			
3	QS (N/kg)	-0.34	0.01*	-1.36 to -0.16	0.76	0.58	0.54
	Ankle dorsiflexion ROM (°)	-0.43	<0.01*	-0.27 to -0.08			
	Mini-BEST (score)	-0.31	0.02*	-0.34 to -0.03			

## Discussion

The results of our study suggest that knee extension muscle strength, ankle dorsiflexion ROM, and balance ability were factors that influence gait variability for inpatients in the clinic, and these three factors could be used as predictors of gait variability. To our knowledge, this is the first trial to examine factors affecting stride time CV for older inpatients.

Our findings are partially consistent with those obtained in previous studies of older adults. Akimoto et al. found that the stride time CV was 2.1 ± 0.7% in healthy older adults and 3.2 ± 1.5% in patients with orthopedic disease [19,20]. Similar results were obtained in our results. Although patients with traumatic fractures at various sites were involved in our study, there was no significant difference in stride time CV between those with various sites of fracture, indicating that stride time CV is not influenced by the site of the fracture and could be representative of gait variability in the orthopedic disease population. Meanwhile, the stride time CV values of the inpatients were also similar to those of the fall population in the previous study [8], suggesting that our subjects had the characteristics of the fall population, according to that almost all participants had also experienced fall history in this study.

In addition, increased gait variability in older people is associated with various factors such as functional status and physical ability [6], while the study by Matsuda et al. [21] and Bogen et al. [22] indicates that knee extension strength and balance are the main influences factors of gait variability specifically. This finding is also consistent with the results of our multiple regression analysis. However, ankle dorsiflexion mobility was one of the factors influencing gait variability, which has been rarely investigated in previous studies. Previous studies have focused on the relationship between the dynamic joint range of motion and gait variability during walking using a motion capture system [23]. Although the devices and calculations differ from our study and cannot be compared, it was established that the ankle range of motion in the sagittal plane is associated with gait variability. On the other hand, proprioception is an important factor in maintaining dynamic balance and postural stability [24,25], and limitations in ankle mobility are associated with the lack of proprioception [26]. We speculate that the lack of proprioception disrupts the walking rhythm at each step, resulting in increased gait variability. These factors may be the reasons why ankle dorsiflexion mobility is influential in gait variability.

According to our results, the balance in model 3 (R2=0.54) did not play a significant role in predicting gait variability compared to the other two predictors in model 2 (R2=0.47). This illustrates that it could be simpler and more effective to combine lower extremity knee extension strength and ankle dorsiflexion mobility than to perform balance training only in clinical treatment when gait improvement is desired. In previous studies, it has been demonstrated that exercise interventions for lower extremity knee extension strength in older adults resulted in a significant reduction in stride time CV [21] and a significant improvement in gait variability. In addition, a simple, time-saving, and effective treatment method is preferred for therapists in clinical treatment. Training focusing on knee extension strength and ankle dorsiflexion mobility not only has a direct impact on balance improvement [27,28] but also has a significant clinical benefit [21,29]. Consequently, improving knee extensor strength and ankle dorsiflexion mobility might be a promising option in the treatment of inpatients to reduce fall risk.

There are several limitations of our study. First, there is an age difference between the inpatients and control groups, implying that age has some interference with gait and various clinical indicators. However, a study found no significant differences in CV and other functional measures of gait between healthy older adults and younger adults, which means that age did not have a significant effect on gait variability [8]. Moreover, the results of our multiple regression analysis showed that the potential prediction of age on gait variability was not as strong as the effects of other factors. Thus, we believe that age does not play a critical role in gait variability. Secondly, our inpatients consist of patients with various types of orthopedic diseases, which is too fragmented in terms of disease types and has some impact on the outcome. Third, the sample size of this study is very small, which may affect the results. Fourth, we used five consecutive strides to calculate gait variability, while more consecutive strides are required for short walks to ensure reliability [30]. Finally, due to the cross-sectional study design of this study, the results did not reveal a causal relationship between gait variability and the influencing factors. Thus, a longitudinal study design is needed to further determine the relationship between gait variability and the influencing factors.

## Conclusions

This study demonstrated that gait variability in older inpatients is influenced by knee extension strength, ankle dorsiflexion mobility, and balance. In addition, we found that quadriceps muscle strength and ankle dorsiflexion mobility made a critical contribution to gait variability. Predictors of gait variability should be given more attention in clinical rehabilitation and nursing care for older inpatients in the future.
